# Bringing back psychological empowerment in empowerment-oriented leadership: the development of the Psychological Empowerment Leadership Scale (PELS)

**DOI:** 10.3389/fpsyg.2025.1539085

**Published:** 2025-07-22

**Authors:** Carsten C. Schermuly, Mona Algner, Timo Lorenz

**Affiliations:** ^1^Institute for New Work and Coaching, SRH University Berlin, Berlin, Germany; ^2^Department of Psychology, Medical School Berlin, Berlin, Germany

**Keywords:** empowering leadership, psychological empowerment, factorial validity, psychometric quality, automated item-selection, PELS

## Abstract

Empowering leadership has garnered significant attention over the past two decades, driven by the evolving dynamics of organizations. However, current measures of empowering leadership often fail to align with the psychological empowerment construct (Spreitzer, 1995) – the very construct these leadership practices claim to impact – and exhibit some psychometric flaws. To address these issues, we introduce the Psychological Empowerment Leadership Scale (PELS), designed to assess leader behaviors fostering psychological empowerment across six dimensions. We used automated item selection algorithms to ensure high psychometric quality and tested the instrument’s validity and measurement invariance in German and US samples. A second study with two measurement points, assessed the criterion-oriented validity of the PELS. Confirmatory factor analyses support its factorial validity and indicate metric measurement invariance across both countries. The PELS shows strong correlations with psychological empowerment, job satisfaction, and emotional exhaustion, often exceeding previous meta-analyses, thus demonstrating criterion validity. However, its association with innovative behaviors was lower than expected, warranting further research. With only 24 items, the PELS, offers high reliability and stability over time, providing a more efficient tool for assessing empowering leadership and aligning better with contemporary theoretical perspectives. This research refines the conceptualization and assessment of empowering leadership in contemporary organizational contexts.

## Development and cross-cultural validation of the Psychological Empowerment Leadership Scale (PELS)

Empowering leadership has been a prominent construct in both research and practice for more than two decades (Lee et al., 2018). This sustained interest likely stems from changes in the internal and external circumstances of contemporary organizations (Amundsen and Martinsen, 2014). Work trends such as digitalization, globalization, knowledge explosion, and demographic changes (in most Western societies) have created an external environment often summarized by the acronym VUCA: volatility, uncertainty, complexity, and ambiguity (Schermuly et al., 2022a). In response, organizations are increasing internal complexity, for example, by flattening hierarchies and delegating responsibility to a broader employee base (Lee et al., 2018). Addressing problems promptly and competently at their source enables organizations to navigate complexity more swiftly and effectively (Amundsen and Martinsen, 2014). However, these changes necessitate adaptations in leadership processes and styles. As organizational environments rapidly evolve and hierarchies flatten, traditional management styles, such as transactional leadership, which thrives in stable and structured settings (Bass, 1985), become less effective. Considering these modern challenges, empowering leadership has emerged as a particularly suitable approach (Lee et al., 2018).

Empowering leadership involves a unique set of behaviors centered around the concept of sharing power. It is characterized by encouraging employee participation, delegation, autonomy, and the removal of bureaucratic barriers (Joo et al., 2016; Cheong et al., 2019; Schermuly et al., 2022a). While it builds upon concepts from supportive leadership, situational leadership theory (which includes delegating behaviors), individualized leadership, and participative leadership, it extends beyond mere participation (Cheong et al., 2019; Kim et al., 2018). Since empowerment is central to empowering leadership, it is important to clarify this concept early on.

Psychological empowerment is widely accepted as the operational definition of empowerment in work and organizational psychology, as evidenced by multiple meta-analyses (Llorente-Alonso et al., 2024; Mathew and Nair, 2022; Seibert et al., 2011). It comprises four perceptions within the work role: meaning, self-determination, competence, and impact (Spreitzer, 1995). Meaning relates to the congruence between employees’ values, beliefs, and behaviors with their job requirements, highlighting identification with job purposes and goals (Spreitzer, 1995). Self-determination pertains to the autonomy employees have in initiating and regulating job-related actions. Competence, associated with Bandura (1997) concept of self-efficacy, refers to employees’ confidence in their job skills (Spreitzer, 1995). Lastly, impact relates to the extent to which employees feel they can influence significant outcomes in their work environment, whether strategic, administrative, or operational (Spreitzer, 1995). These facets together foster a proactive and intrinsically motivated work orientation, forming the holistic *Gestalt* of employee empowerment (Spreitzer, 1995; Schermuly et al., 2022a).

“The returns of empowering leadership are often claimed to be mostly beneficial, humane, and virtuous” (Cheong et al., 2019, p. 34). This positive appraisal is echoed in the substantial body of research on the subject, including meta-analyses by Kim et al. (2018) or Lee et al. (2018). These studies have demonstrated positive employee reactions to empowering leadership, which prove advantageous not only for organizations but also for employees themselves. When sorted by the magnitude of effects, correlations have been observed with variables such as trust in the leader, Leader-Member Exchange (LMX), perceived leader effectiveness, role clarity, knowledge sharing, work engagement, commitment, creativity and innovative behavior, and performance (Kim et al., 2018). Employees who are committed, engaged, creative, and high-performing are especially valuable in navigating the challenges of VUCA environments.

Surprisingly, the strongest association with empowering leadership is not with psychological empowerment, contrary to what one might expect from a leadership style intended to empower employees (Kim et al., 2018). Meta-analytic findings (Kim et al., 2018) show a quite strong correlation with trust at *r* = 0.57, while the correlation with psychological empowerment is weaker at *r* = 0.41, indicating only a medium-sized effect. Furthermore, some issues arise regarding the validity of empowering leadership, as it does not predict psychological empowerment more effectively than other leadership styles. In a meta-analysis comparing the effects of empowering leadership with transformational, servant, and transactional leadership on psychological empowerment, Schermuly et al. (2022b) found that empowering leadership only showed advantages over transactional leadership, but not over transformational or servant leadership. These findings highlight a need to conceptually realign empowering leadership more closely with psychological empowerment. An instrument that promises empowerment as a target variable should also adequately relate to modern perspectives on empowerment both theoretically and empirically. To address this gap, we have conceptualized and developed a new instrument specifically designed to assess empowering leadership, aiming to better capture its unique characteristics and effectiveness in fostering psychological empowerment. Our instrument is not intended to replace other instruments, but to offer researchers and practitioners an additional alternative.

### Assessing empowering leadership

In contrast to other leadership styles such as transformational leadership [see the Multifactor Leadership Questionnaire; MLQ, Bass and Avolio (1995)], there is no single, dominant measure for empowering leadership. The assessment of empowering leadership is therefore highly inconsistent (Cheong et al., 2019) which is a challenge in empowering leadership research. Cheong et al. (2019) identified different instruments, but the instrument developed by Arnold et al. (2000) (Empowering Leadership Questionnaire; ELQ) seems to be the most popular one. It consists of five different dimensions with 38 items: Coaching (11 items), leading by example (5 items), informing (6 items), participative decision making (6 items) and showing concern (10 items). The ELQ was developed inductively (Amundsen and Martinsen, 2014). Arnold et al. (2000) sought “up-to-date information, to better understand the behaviors required in empowered team environments” (p. 252) through in-depth interviews with team leaders and members from three empowering organizations, rather than deriving dimensions directly from the empowerment literature for example published by Spreitzer.

The procedure in the development process might explain the absence of a direct conceptual link to the four dimensions of psychological empowerment in the ELQ, as the managers were likely unfamiliar with the concept. For example, none of the five dimensions directly relate to the meaning dimension. Another challenge is the predominance of the participation dimension. Several scholars see participation as a construct with varying levels, often considering informing employees as the lowest rung on this escalator (Dachler and Wilpert, 1978; Tannenbaum and Schmidt, 1973; Wilkinson et al., 2010). Accordingly, two of the five dimensions of the ELQ assess participation. But there are other rather methodological limitations, such as the very high intercorrelations between factors. For instance, coaching and participative decision making exhibit such a high correlation (*r* = 0.94) that it raises doubts about treating them as distinct factors. Furthermore, the unequal distribution of items across factors complicates comparisons and introduces challenges in assigning appropriate weights for calculating an overall mean score (Furr, 2011). Finally, with 38 items, this instrument is even longer than the MLQ. While this may bolster reliability, such extensive length can also impede practical utility.

In contrast, for example Konczak et al. (2000) developed a six factor instrument with fewer items (17) which they called Leader Empowering Behavior Questionnaire (LEBQ). This instrument consists of the following dimensions: Accountability (3 items), coaching for innovative performance (3 items), delegation of authority (3 items), information sharing (2 items), self-directed decision making (3 items), skill development (3 items). Again, leader behaviors that directly target the dimension of meaning within the psychological empowerment construct are absent. Additionally, the dimension of accountability does not appear to clearly align with fostering empowerment-related cognitions and feelings. Items such as “My manager holds people in the department accountable for customer satisfaction” or “I am held accountable for performance and results” reflect a transactional approach. In a modern VUCA workplace, where customer satisfaction or performance often depend on collective efforts rather than individual actions, these items may not effectively capture the construct of psychological empowerment. This issue seemed to be already evident in the results published in the original article (Konczak et al., 2000). While psychological empowerment correlated strongly with the dimension delegation of authority (*r* = 0.62), the correlation with accountability was much weaker (*r* = 0.23). Despite these findings, the accountability dimension was integrated into the questionnaire and empowering leadership continues to be assessed in this manner today. Additionally, the discriminant validity of the LEBQ was not tested, and there are challenges related to item formulation. Specifically, there is a discrepancy in the level of analysis: most items assess the manager (“My manager”), while some also focus on the individual-level perceptions (“I am held accountable”). Furthermore, certain items do not reflect a proactive form of leader behavior but rather a laissez-faire attitude of leadership (e.g., “My manager relies on me to make my own decisions about issues that affect how work gets done”).

The newest scale is the Empowerment Leadership Scale (ELS; Amundsen and Martinsen, 2014) and even this instrument does not theoretically seek close contact with all dimensions of psychological empowerment. This instrument comprises only two dimensions. Autonomy Support (12 items) directly addresses the self-determination aspect, and Development Support (6 items) focuses on the competence dimension within the empowerment construct. However, this scale does not include dimensions assessing the behaviors of leaders fostering meaning and impact.

### Goals for a new instrument

The primary goal of our new tool is to embed it more strongly in current empowerment research. Conceptually, the scale is designed to assess managers’ empowerment-promoting behaviors across the four dimensions of psychological empowerment. The aim is not to assess the psychological empowerment of managers or employees, but rather the behaviors of supervisors that can promote the psychological empowerment of employees. Furthermore, we aim to enhance the content validity of our measurement tool by ensuring comprehensive coverage of the empowering leadership construct, thereby providing a more holistic assessment of empowerment-promoting behaviors. This approach addresses limitations of previous instruments that may have overlooked certain facets of the construct, thus improving the validity and applicability of our scale in both research and practice.

Our second goal is to assess empowering leadership with a contemporary set of items. To ensure that empowering leadership remains relevant in practice, it is essential to provide an updated measurement tool. To accomplish this and reach a high psychometric quality, we employ automated item selection algorithms (e.g., genetic algorithms, ant colony optimization algorithms; Galán et al., 2013; Schultze, 2017). These meta-heuristics aid in creating psychometrically sound assessment tools, by leveraging machine learning algorithms. Despite their effectiveness, these algorithms are not yet widely adopted in organizational science [see Algner and Lorenz (2022), Pundt et al. (2022), and Schneider et al. (2024), for examples of their use]. With this article, we aim to demonstrate how scholars can use machine learning algorithms to improve scale construction efforts.

## Dimensions of the new instrument

### Sense making

“Communicating purpose is the most central of all leader behaviors, because it infuses work with meaning and direction” (Carton et al., 2014, p. 1555). Supervisors help employees to understand the meaning of their work, thereby providing direction and orientation (Schermuly, 2024). This first dimension of the PELS specifically targets and promotes the experience of meaning at work and thus the meaning dimension in Spreitzer (1995) empowerment concept. We define sense making as leader behaviors that aim to stimulate the experience of job meaning on the part of employees.

This central perspective and orientation toward the standard empowerment concept is missing in the existing questionnaires such as those by Konczak et al. (2000), Arnold et al. (2000), or Amundsen and Martinsen (2014). However, a similar dimension can be found in other leadership concepts. In purpose-driven leadership (PDL) supervisors try to find their personal purpose, assist employees in finding their own purpose and integrate the personal with the organizational purpose (Cardona et al., 2019). Additionally, there is an association with the transformational leadership dimension of inspirational motivation, where leaders articulate a desirable vision for the future and help to find ways to reach it (Bass, 1999). The difference lies in empowerment-oriented leaders stimulating meaning not solely through a vision but also through daily actions, actively creating meaning in their leadership approach.

Several specific leadership behaviors fall within this dimension. Supervisors fulfill their role as the stimulator of meaning by explaining the purpose behind actions within the department or organization. They assist employees in understanding why their work is meaningful, not only for themselves but also for the department, organization, or sometimes for society (greater good motivation; Steger et al., 2012). Leaders provide employees with an interesting outlook on their work in the future, but also ensure that they have as few meaningless tasks as possible (Schermuly, 2024).

### Competence development

The second dimension directly linked to Spreitzer (1995) empowerment concept is competence development. This involves managers proactively facilitating the growth of job-related competencies within their team, rather than relying solely on HR departments for training. Therefore, competence development includes the leader activities to support the improvement of skills and self-efficacy of the employees. Regarding Spreitzer (1995) empowerment concept, we consider both factors to be important. Not only should competencies be developed, but employees should also develop confidence in these competences.

A similar dimension in the LEBQ (Konczak et al., 2000) is referred to as skill development. However, there are conceptual differences between this and the competence development dimension in PELS. Konczak et al. (2000) do not explicitly define the dimension but the focus of the three items suggests it is primarily concerned with fostering competencies rather than supporting the experience of competence. At the same time, some of the items are formulated very broadly (e.g., “My manager ensures that continuous learning and skill development are priorities in our department”) while others include very specific examples (e.g., “My manager encourages me to use systematic problem-solving methods, e.g., the seven-step problem-solving model”).

In our approach aligned with the empowerment concept, this dimension should emphasize direct and concrete interactions between supervisors and employees. We therefore focus on specific behaviors, such as managers helping employees learn from mistakes, providing regular feedback, and encouraging employees to reconsider job-related issues. In addition, we include behaviors such as praising skills or expressing appreciation for an employee’s abilities.

### Participation

The third dimension of the PELS is participation. Participative leader behaviors target especially the self-determination dimension of the psychological empowerment model. There are many different definitions and conceptualizations of participative leadership, “but its main focus has always been on the individual subordinate’s or group’s participation in decision making that would normally be done by the leader in a classically structured, hierarchical organization” (Kim et al., 2018, p. 258).

Given its central role in empowering leadership, this dimension appears in all previous instruments: informing and participative decision making (ELQ), self-directed decision making (LEBQ), autonomy support (ELS). It is also the dimension with the longest history: Since the experiments by Lewin et al. (1939) on the different effects of participative, autocratic and laissez-faire oriented leadership, participative leadership has been a prominent research topic in organizational research. Several meta-analyses show the positive effects of participative leadership on variables such as satisfaction and productivity, particularly when complex work tasks have to be handled and processed (Miller and Monge, 1986; Gastil, 1994). Participative leadership is part of different theoretical models such as proposed by Tannenbaum and Schmidt (1973) or Vroom and Yetton (1973).

In our operationalization, various leadership behaviors belong to this dimension. These include, for example, providing employees with information at an early stage, explaining decisions, seeking advice on important decisions and giving employees complete autonomy about relevant work processes.

### Transfer of power

We call the fourth dimension of the PELS the transfer of power. In Spreitzer (1995) empowerment concept, this dimension aims to capture the employees’ experience of impact. Conger and Kanungo (1988) have previously defined empowerment as “the process by which a leader or manager shares his or her power with subordinates” (p. 473).

Neither the ELQ nor the ELS includes a dimension specifically addressing power. The lack of a power dimension is surprising, given the foundational role of power sharing in the empowerment concept, the early literature’s emphasis on this aspect, and the linguistic proximity of power-related constructs to empowerment. Even in the LEBQ, the dimension called “delegating authority” falls short of capturing the full scope intended in the PELS. Our dimension aims to assess whether managers actively share their power with employees. According to Tost (2015) “Power refers to asymmetric control over valued resources, which in turn affords an individual the ability to control others’ outcomes, experiences, or behaviors” (p. 30). Power is thus a relational construct involving at least two individuals (Conger and Kanungo, 1988). In hierarchical organizations, supervisors typically hold most of the control over resources such as budgets, career paths, and information. Power sharing not only benefits societies (e.g., stability and peace, or minimizing division; Farag et al., 2023), but also enhances job performance within organizations through psychological empowerment (Chen et al., 2014).

Empowering leaders share their power with their subordinates and delegate authority (Conger and Kanungo, 1988). Rather than controlling their employees, they provide resources and support to help employees influence work objectives and achieve their goals.

### Coaching

So far, we have introduced four dimensions of empowering leadership that are directly related to the four facets of psychological empowerment. However, other, more global leadership behaviors might also stimulate psychological empowerment. In both the ELQ and the LEBQ, a dimension called coaching focuses on the leader’s relationship work and support for employees (e.g., “Provides help to work group members” or “Helps develop good relations among work group members“; Arnold et al., 2000, p. 269). Several studies show that relationship work can foster psychological empowerment (see, for example, LMX research and the meta-analysis by Dulebohn et al., 2012). To maintain continuity with previous empowerment assessments, we adopt and operationalize this dimension as coaching. However, we also recognize parallels with the construct of individual consideration in the Ohio State Leadership Studies (Judge et al., 2004).

It is important to highlight a fundamental difference between the role of a supervisor demonstrating coaching behaviors and that of an external coach. While supervisors engaged in coaching behaviors by investing time to understand and discuss employees’ concerns, they retain their supervisory role and do not assume the role of a coach. Unlike supervisors, external coaches work on an equal footing with their clients, without a disciplinary function (Grant and Stober, 2006; Graßmann et al., 2020).

### Leading by example

The final dimension is leading by example. This dimension has also been emphasized in the ELQ as an important part of the empowering leadership process. Leading by example refers to a supervisor displaying behaviors “that show the leader’s commitment to his or her own work as well as the work of his/her team members” (Arnold et al., 2000, p. 254). Idealized influence is defined in the literature as “the capability of exerting influence by serving as a role model, demonstrating high performance as well as moral standards” (Felfe et al., 2004, p. 267).

In organizations, supervisors serve as role models for appropriate behaviors and values (Ambrose et al., 2013) and are often imitated by their team members (Lu et al., 2018). Research has shown that social learning between supervisor and employees is important for the empowering process (Grützmacher and Schermuly, 2021). Supervisor demonstrating low commitment toward their work, working less than their employees and withdrawing during busy periods are likely to disempower their team members. On the other hand, supervisors who exemplify a high work ethic and, for example, also experience meaning in their work tasks should empower their employees. Having detailed the theoretical foundation of our new tool, we will now outline our approach to validating it.

### Validation strategy

We use various approaches to validate the questionnaire. To assess convergent validity, we use confirmatory factor analysis (CFA) and compare the PELS with established instruments such as the Empowering Leadership Questionnaire (ELQ) and the Empowerment Leadership Scale (ELS). If the PELS is convergent valid, then it should correlate with these instruments. So, we postulate:

*H1:* PELS is positively correlated with ELQ and ELS

To test criterion validity, we examine the correlations between the PELS and various variables which are established in empowering leadership research. We categorize these variables into two groups: cognitively and emotionally oriented variables (e.g., psychological empowerment, job satisfaction, emotional exhaustion) as well as more performance-related variables (e.g., performance and innovation behavior).

The target construct of empowering leadership is psychological empowerment, as conceptualized by Spreitzer (1995). The behaviors are designed to increase the psychological empowerment of employees as explained above. Empirical evidence supports that empowering leadership is positively associated with psychological empowerment, as demonstrated in meta-analyses (Schermuly et al., 2022a). Therefore, we propose the following hypothesis:

*H2:* PELS correlates positively with psychological empowerment

Kim et al. (2018) show meta-analytically that empowering leadership is associated with reduced negative emotions. They explain this with the fact that people who are led by empowering leaders experience more positive work situations. According to the conservation of resources theory (Hobfoll, 2011), employees who are led in an empowerment-oriented manner have more resources and, therefore, more energy in their day-to-day work. For example, high job demands are perceived as less exhausting if employees experience control over their work (Bakker and Demerouti, 2007). That is why we postulate:

*H3:* PELS correlates positively with job satisfaction

*H4:* PELS correlates negatively with emotional exhaustion

In the same meta-analysis, Kim et al. (2018) also report correlations between empowering leadership and innovation behavior as well as performance. People who work with empowering supervisors should be more motivated but also more proactive (Spreitzer, 1995). For example, the creation of meaning should stimulate intrinsic motivation. By giving employees more freedom and autonomy, they should have the opportunity to try out and implement more ideas (Schermuly et al., 2013). Therefore, we postulate:

*H5:* PELS correlates positively with work performance

*H6:* PELS correlates positively with innovative behavior

As all correlations have already been well documented in meta-analyses, we have provided only a brief theoretical rationale for our hypotheses.

## Method Study 1

### Procedure

The primary objective of the first study was to develop and validate the PELS. Germany and the United States were selected as study contexts because they differ in key cultural dimensions relevant to leadership and empowerment processes. For example, the U. S. typically scores lower on power distance and higher on individualism compared to Germany (Hanges and Gupta, 2004), potentially affecting how leadership behaviors are interpreted. Including both countries therefore allowed us to test whether the PELS functions equivalently across culturally distinct Western environments, strengthening the cross-cultural validity and applicability of the instrument. Furthermore, validation of the instrument in the U. S. is important because the construct originated there and there are many users in research and practice.

Accordingly, we recruited two samples of employees from Germany and the USA. We used a two-stage approach. In the first stage, we developed a large item pool that was administered to all participants. Next, we utilized a genetic algorithm approach in the German sample to select the items. In the second stage, we validated the selected items using the US sample.

We collected the US data via an online panel provider (i.e., Prolific). For the German survey, participants were recruited via personal and professional networks as well as several online social media platforms. To ensure high data quality and limit insufficient effort responding in our survey, we followed best practices (e.g., Aguinis et al., 2021; Curran, 2016). For instance, we conducted a soft launch of our studies with 10 participants to ensure clarity and to preempt any potential technical issues. To assess insufficient effort responding, we incorporated three instructed response items. Participants were asked to respond according to specific instructions (e.g., “Please select answer option agree”). Participants who failed to answer at least two out of the three items correctly were excluded from the analysis. We also investigated whether any participants completed the survey exceptionally quickly. We applied a criterion of <2 s per item, as previous research has suggested that this time frame is too brief for participants to fully read and comprehend each item (Ward and Meade, 2023). In the US sample, no participants were excluded. In the German sample, nine individuals (2.4%) were removed from the final dataset because they failed the attention checks.

For the US sample, we employed *Prolifics* screening criteria, inviting only participants with a high approval rate (i.e., 95% or higher across a minimum of 100 studies). Each participant received £1.8 compensation for 12 min of survey time (equivalent to £9 per hour). This compensation was carefully calibrated to encourage participation without excessively incentivizing it.

### Participants

#### US sample

The US sample consisted of 290 participants with a mean age of 41.1 years (*SD* = 12.0), including 47.2% men, 51.0% women, and 1.0% non-binary individuals. Participants reported working an average of 38.1 h per week (*SD* = 7.3) and had been with their current organization for an average of 7.0 years (*SD* = 6.4). They worked with their current leader for an average of 4.5 years (*SD* = 4.1). The median organization size was reported as 300 employees, with teams typically composed of seven members. Leaders were predominantly men (56.2%) with 43.8% women leaders. Participants worked in various sectors, with the three largest shares being 14.5% in health and social services, 12.4% in education, and 21.4% in other sectors. These sectors include both public and private organizations, and span knowledge-intensive, service-oriented, and regulated industries. The sample includes participants in professional, technical, and managerial roles, providing insight into a broad range of employee-leader relationships in contemporary workplaces. A majority (67.6%) held a college degree or higher. Employment roles included 65.2% employees and 30.3% in leadership positions, with 19% working in more than one team.

#### German sample

The German sample included 362 participants, averaging 40.3 years old (*SD* = 10.57), with 27.6% men, 72.1% women, and 0.3% non-binary individuals. These participants worked an average of 36.4 h per week (*SD* = 6.0), had been part of their current organization for 7.9 years (*SD* = 8.9), and had worked under their current leader for 3.2 years (*SD* = 3.1). Organizations had a median size of 1,000 employees, and teams a median size of 8 members. Leaders of the participants were 66% men and 34% women. Participants worked in various industries, with the three largest sectors being 13.0% in manufacturing, 10.5% in research and development, and 10.2% in consulting. These industry sectors reflect a broad cross-section of the German workforce, including both production-oriented and knowledge-intensive environments. Participants held diverse roles, with a strong representation of professionals and middle managers working within complex organizational structures. The sample exhibited a high level of education, with 82.6% having a college degree or higher. In terms of roles, 61.1% were employees and 32.6% held leadership responsibilities. Furthermore, 59.1% worked in more than one team.

### Materials

#### Demographics

Participants were asked to provide demographic and work-related information, including their age, gender, highest level of education, position in the organization, team size, employment status, size of organization, age and gender of their leader, duration of working with the leader, weekly working hours, total years of work experience, and sector of employment.

#### Empowering leadership

##### Empowering leadership questionnaire

Empowering Leadership was assessed using the Empowering Leadership Questionnaire (ELQ; Arnold et al., 2000). Participants were asked to rate how much they agree with statements such as “My manager works as hard as they can.” Answer scales ranged from 1 (*Never*) to 5 (*Always*). Cronbach’s alpha was 0.98 in the German sample and 0.98 in the US sample. McDonald’s omega ω*_t_* was 0.98 in the German sample and 0.99 in the US sample.

##### Psychological Empowerment Leadership Scale (PELS)

The newly developed Psychological Empowerment Leadership Scale (PELS) was used to assess Empowering Leadership. On a scale from 1 (*I do not agree at all*) to 7 (*I fully agree*), participants rated 64 items such as “My manager explains to me why my work matters” or “My manager provides me with important information I need for my work in a timely manner.” For details on the final reduced item set, as well as reliability statistics including Cronbach’s alpha and McDonald’s Omega ω*_t_*, refer to Table 1.

**Table 1:** Final item list of the Psychological Empowerment Leadership Scale and factor loadings.

Sub facet	German Wording	English wording	Standardized factor loading		Standard error	
GER/US	Meine Führungskraft…	My leader…	GER	US	GER	US
*Sense making* *α* = 89/0.95 *ω* = 0.90/0.95	vermittelt mir eine interessante Perspektive auf meine Arbeit in der Zukunft.	provides me with an interesting perspective on my work in the future.	0.841	0.883		
	erarbeitet mit mir eine Vision für meine Arbeit.	works with me to develop a vision for my work.	0.859	0.904	0.048	0.043
	hilft mir, in meiner Karriere Sinn zu erleben.	helps me to experience a sense of meaning in my career.	0.917	0.943	0.050	0.039
	unterstützt mich zu erkennen, welchen Wert meine Arbeit für die Gesellschaft besitzt.	supports me in recognizing the value of my work for society.	0.686	0.886	0.056	0.039
*Coaching* *α* = 0.93/0.91 *ω* = 0.93/0.91	behandelt mich als Individuum.	treats me as an individual.	0.875	0.865		
	interessiert sich dafür, wie es mir geht.	is interested in how I am feeling.	0.918	0.905	0.050	0.055
	nimmt sich Zeit, um meine Anliegen zu verstehen und zu diskutieren.	takes the time to understand and discuss matters of my colleagues and I.	0.880	0.909	0.051	0.056
	behandelt mich nicht als einen Mitarbeitenden unter vielen.	does not treat me as one employee among many.	0.828	0.710	0.047	0.070
*Participation* *α* = 0.85/0.91 *ω* = 0.86/0.91	versorgt mich rechtzeitig mit wichtigen Informationen, die ich für meine Arbeit	provides me with important information I need for my work in a timely manner.	0.747	0.857		
	erklärt ihre Entscheidungen.	explains their decisions.	0.842	0.893	0.069	0.049
	holt meinen Rat bei wichtigen Entscheidungen ein und berücksichtigt ihn.	seeks and considers my advice on important decisions.	0.712	0.776	0.069	0.067
	erläutert den Mitarbeitenden die Entscheidungen des Unternehmens.	explains decisions of the organization to the employees.	0.787	0.854	0.077	0.054
*Transfer of power* *α* = 0.85/0.89 *ω* = 0.86/0.89	kontrolliert mich nicht.	does not control me.	0.584	0.669		
	ermutigt mich, eigene Wege zu finden, um Arbeitsprobleme zu lösen.	encourages me to find my own ways to solve work problems.	0.829	0.789	0.138	0.091
	teilt mit den Mitarbeitenden Macht.	shares power with the employees.	0.837	0.871	0.139	0.126
	ermöglicht mir, dass meine Arbeit Einfluss auf die Zielerreichung meines Teams hat.	enables my work to have an impact on my team’s goal achievement.	0.822	0.909	0.124	0.133
*Competence development* *α* = 0.87/0.94 *ω* = 0.88/0.95	hilft mir, meine beruflichen Fähigkeiten weiterzuentwickeln.	helps me to develop my professional skills.	0.860	0.928		
	hilft mir, mich auf die Position im Unternehmen vorzubereiten, die ich wirklich haben will.	helps me prepare for the position in the organization that I really want.	0.841	0.919	0.037	0.027
	regt mich zum Nachdenken über berufliche Probleme an.	encourages me to think about professional problems.	0.743	0.895	0.050	0.036
	lobt mich, wenn ich meine Kompetenzen verbessere.	praises me when I improve my skills.	0.754	0.865	0.054	0.040
*Leading by example* *α* = 0.90/0.96 *ω* = 0.90/0.96	Zeigt bei ihrer Arbeit genauso hohen Einsatz wie ihre Mitarbeitenden.	Shows the same high level of commitment to their work as their employees.	0.869	0.923		
	wird ihren eigenen hohen Standards selbst gerecht.	lives up to their own high standards.	0.847	0.966	0.056	0.033
	zieht sich nicht zurück, wenn es mal mehr zu tun gibt.	does not withdraw when there is more work to do.	0.728	0.910	0.047	0.039
	lebt eine hohe Arbeitsmoral vor.	exemplifies a high work ethic.	0.864	0.915	0.056	0.048

#### Psychological empowerment

Psychological empowerment was assessed using Spreitzer (1995) questionnaire. Participants were asked to rate how much they agree with statements such as “The work I do is meaningful to me.” Answer scales ranged from 1 (*Strongly disagree*) to 7 (*Strongly agree*). Cronbach’s alpha was 0.89 in the German sample and 0.95 in the US sample. McDonald’s omega ω*_t_* was 0.89 in the German sample and 0.95 in the US sample.

#### Job satisfaction

Job satisfaction was assessed using three items from the Job Diagnostic Survey (Hackman and Oldham, 1975). Participants were asked to rate how much they agree with statements such as “Generally speaking, I am very satisfied with this job.” Answer scales ranged from 1 (*Strongly disagree*) to 7 (*Strongly agree*). Cronbach’s alpha was 0.82 in the German sample and 0.88 in the US sample. McDonald’s omega ω*_t_* was 0.83 in the German sample and 0.89 in the US sample.

#### Emotional exhaustion

Emotional exhaustion was assessed using the emotional exhaustion items of the Oldenburg Burnout Inventory (OLBI; Demerouti and Nachreiner, 1998). Participants were asked to rate how much they agree with statements such as “There are days when I feel tired before I arrive at work.” Answer scales ranged from 1 (*Strongly disagree*) to 4 (*Strongly agree*). Cronbach’s alpha was 0.85 in the German sample and 0.92 in the US sample. McDonald’s omega ω*_t_* was 0.85 in the German sample and 0.92 in the US sample.

#### Job performance

Job performance was assessed with the Individual Work Performance Questionnaire (Koopmans et al., 2014). Participants were asked to rate how much they agree with statements such as “In the past four weeks I took on extra responsibilities.” Answer scales ranged from 1 (*Never*) to 5 (*Always*). Cronbach’s alpha was 0.80 in the German sample and 0.92 in the US sample. McDonald’s omega ω*_t_* was 0.80 in the German sample and 0.92 in the US sample.

### Data analysis

We developed the PELS using an automated item selection algorithm based on metaheuristics. Given the limited adoption of algorithmic approaches in organizational and social sciences, we provide a concise overview of the procedure [for an extensive introduction to metaheuristics, particularly genetic algorithms, refer to Gendreau and Potvin, 2010 and Reeves, 2010]. The process of scale development involves selecting items to construct a psychometrically sound scale and can be conceptualized as a combinatorial problem (Kerber et al., 2022). Combinatorial problems, such as the knapsack problem (Schroeders et al., 2016), entail finding a discrete solution within specified constraints (Hoos and Stützle, 2004). Although traditionally associated with economics, these problems have been recently applied to item selection in psychological scale construction (e.g., Schultze, 2017; Kerber et al., 2022) to form sets of items meeting predefined criteria (e.g., constructing a two-dimensional scale with favorable model fit). Using metaheuristic algorithms for item selection offers the advantage that they can efficiently search large solution spaces to identify optimal or near-optimal sets of items, which can be particularly useful when dealing with complex criteria and constraints (e.g., finding the best combination of items to capture a psychological construct).

Contemporary methodologies employ automatic optimization algorithms like Genetic Algorithms (GA; Holland, 1992), rooted in natural evolution, to address combinatorial problems. In contrast to classical approaches, heuristic item selection algorithms, such as GAs, aim to enhance psychometric properties within defined constraints (Schultze, 2017). A key characteristic is the approximate, rather than deterministic, nature of metaheuristics (Blum and Roli, 2003), as they acknowledge the difficulty of finding the single-best solution (Yarkoni, 2010). Nevertheless, approximate algorithms, such as metaheuristics, are crucial for achieving near-optimal solutions in complex combinatorial problems efficiently (Dorigo and Stützle, 2010). Notably, these algorithms excel in considering psychometric criteria in conjunction with other items to enhance the overall scale quality (Olaru and Danner, 2021). Recent research indicates that algorithmic approaches perform on par with or even outperform traditional methods in scale development (Sandy et al., 2014; Schroeders et al., 2016; Olaru and Danner, 2021).

#### Item selection procedure

In this study, we utilized a genetic algorithm to select 24 items for the final version of the PELS. Genetic algorithms aim to streamline a large set of variables through stochastic search methods inspired by evolutionary processes, where the quality of a solution, its fitness, determines its likelihood of survival and reproduction (Galán et al., 2013). These algorithms involve both variation and selection processes to balance diversity and quality, ultimately generating an optimal or near-optimal solution (Galán et al., 2013). Applied to scale development, the algorithm initiates with genes representing parameters or variables, which are organized into chromosomes representing sets of items or scales. An initial population is generated by randomly creating a predefined number of chromosomes (typically 100–200) from the original item pool to ensure variability (Yarkoni, 2010). The fitness function is crucial as it defines the quality of a solution based on psychometric properties. In each generation, the fittest chromosomes (i.e., those with the highest fitness scores) are selected to propagate and serve as the foundation for subsequent generations. Genetic diversity is maintained through mutation and recombination processes. After a predetermined number of iterations (e.g., 100+), the fittest chromosome is typically identified as the optimal solution.

We utilized a genetic algorithm implemented in the R package stuart (v0.9.1; Schultze, 2017) to construct a six-dimensional scale with 24 items. Solutions were assessed against an objective function comprising standard model fit criteria such as the Root Mean Square Error of Approximation (RMSEA), Standardized Root Mean Square Residual (SRMR) and Comparative Fit Index (CFI), as well as composite reliability (McDonald’s *ω*).

#### Evaluation of model fit, measurement invariance, and external validity

Model fit was evaluated following recommendations by Hu and Bentler (1999). They comprised a SRMR ≤ 0.08 in combination with at least one of the following fit indices: a RMSEA ≤ 0.06, a lower bound of the 90% confidence interval (CI) of the RMSEA ≤ 0.06, a CFI ≥ 0.95, or a Tucker-Lewis Index (TLI) ≥ 0.95. Confirmatory factor analysis (CFA) was conducted using the R package lavaan (Rosseel, 2012).

We conducted multi-group CFAs comparing participants from Germany and the US to assess measurement invariance at four hierarchical levels using the R packages lavaan (v0.6–15; Rosseel, 2012) and semTools (v0.5–6; Jorgensen et al., 2022). We estimated four models corresponding to the four hierarchical levels of measurement invariance (Meredith, 1993): configural, metric, scalar, and strict. Configural invariance ensures the same factorial structure across groups (Luong and Flake, 2023), while metric invariance requires equal factor loadings, facilitating comparisons of correlations (French and Finch, 2016). Scalar invariance imposes equality constraints on item intercepts, enabling comparisons of means (Chen, 2007; Luong and Flake, 2023), and strict invariance extends this equality to residual variances, indicating identical item-level measurement (Luong and Flake, 2023) These levels of invariance are critical for valid cross-cultural comparisons in psychological research.

To assess our findings, we estimated the difference in chi-square as well as in CFI and RMSEA across increasingly constrained model specifications. Following guidelines from Putnick and Bornstein (2016) and Chen (2007), changes <0.01 in CFI and <0.015 in RMSEA indicate acceptable relative fit.

## Results Study 1

### Confirmatory factor analyses

The measurement models for Study 1 indicated acceptable to good fit across various scales and samples, using the MLR estimator. In the German sample, the newly developed PELS demonstrated a CFI and TLI of 0.96, and an RMSEA of 0.053, signifying a good model fit. The US sample presented similar fit indices for the PELS, with a CFI and TLI of 0.96 and an RMSEA of 0.065.

For the ELQ, the German sample showed borderline acceptable fit indices with a CFI and TLI of 0.90 and an RMSEA of 0.071, indicating that while the model fit is on the edge of acceptability, there is room for improvement. The US sample exhibited slightly better fit indices for the ELQ, with a CFI of 0.92, a TLI of 0.91, and an RMSEA of 0.076, suggesting that the model fits the data moderately well in both samples, though the US sample showed a marginally better fit. An overview of all measurement models in Study 1 is given in Table 2.

**Table 2:** Measurement models of Study 1 using MLR^a^ estimator.

Variable	*N* factors	*Χ*^2^	df	*p*	CFI	TLI	SRMR	RMSEA	RMSEA 90% CI
PELS									
GER	6 + g	448.37	246	<0.001	0.96	0.96	0.039	0.053	0.045–0.061
US	6 + g	469.70	246	<0.001	0.96	0.96	0.040	0.065	0.056–0.074
ELQ									
GER	5 + g	1714.69	660	<0.001	0.90	0.90	0.052	0.071	0.067–0.075
US	5 + g	1466.85	660	<0.001	0.92	0.91	0.047	0.076	0.071–0.082
Empowerment									
GER	4 + g	101.10	50	<0.001	0.98	0.98	0.048	0.055	0.040–0.071
US	4 + g	85.82	50	0.001	0.99	0.99	0.039	0.057	0.036–0.77
Emotional exhaustion									
GER	1	0.28	2	0.869	1.00	1.01	0.004	0.000	0.000–0.067
US	1	3.66	2	0.161	1.00	0.99	0.010	0.069	0.000–0.179
Job performance									
GER	1	233.22	20	<0.001	0.67	0.54	0.094	0.201	0.178–0.225
US	1	117.79	20	<0.001	0.90	0.86	0.042	0.161	0.133–0.189

*N* = 652; Germany *n* = 362; US *n* = 290.

^a^Maximum likelihood with robust standard errors; PELS, Psychological Empowerment Leadership Scale; ELQ, empowering leadership questionnaire.

### Measurement invariance

We conducted measurement invariance analyses for the PELS across German and US samples using multi-group CFAs (see Table 3). Configural invariance was established with good model fit (CFI = 0.95, RMSE*A* = 0.066, SRMR = 0.039). This indicates that the same latent factor structure holds across both groups. We further found support for metric invariance (ΔCFI = −0.002, ΔSRMR = 0.015), with good model fit (CFI = 0.95, RMSE*A* = 0.066, SRMR = 0.055), suggesting that the strength of relations between latent variables and their indicators is comparable across cultures (Chen, 2007). This means that researchers can compare correlations and regression coefficients between German and US samples.

**Table 3:** Results of measurement invariance analyses for the PELS in the German and the US samples.

Model	*χ*^2^ (df)	CFI	TLI	RMSEA [90% CI]	SRMR	AIC	BIC	Model comp	Δ*χ*^2^ (Δdf)^a^	*p* ^b^	ΔCFI	ΔRMSEA	ΔSRMR	Decision
Configural	1195.1 (492)***	0.95	0.95	0.066 [0.062, 0.071]	0.039	49367.11	50066.00							Accepted
Metric	1243.2 (515)***	0.95	0.95	0.066 [0.062, 0.071]	0.055	49369.22	49965.06	Configural	48.11 (23)	0.002	−0.002	0.000	0.015	Accepted
Scalar	1415.8 (532)***	0.94	0.94	0.071 [0.067, 0.075]	0.059	49507.74	50027.42	Metric	172.52 (17)	<0.001	−0.010	0.006	0.005	(Borderline) accepted
Strict	1804.9 (556)***	0.92	0.92	0.083 [0.079, 0.088]	0.058	49848.89	50261.05	Scalar	389.15 (24)	<0.001	−0.024	0.012	−0.001	Rejected

*N* = 652; Germany *n* = 362; US *n* = 290.

^a^Chi-square difference test refers to standard (rather than to robust) test statistics and was Satorra-Bentler scaled (see Satorra and Bentler, 2001). ^b^*p*-value corresponds to chi-square difference test.

^*^p ≤ 0.05; **p ≤ 0.01; ***p ≤ 0.001.

Scalar invariance was borderline accepted with ΔCFI = −0.010, ΔRMSE*A* = 0.006, ΔSRMR = 0.005. Model fit was still acceptable (CFI = 0.94, RMSE*A* = 0.071, SRMR = 0.059). These results indicate that in addition to ensuring the factor structure and factor loadings are equivalent across groups, item intercepts are equal as well. This ensures that comparisons of observed or latent scores between different cultural or demographic groups are unbiased (Chen, 2007; Luong and Flake, 2023). Strict invariance, which requires equality not only in factor structure, factor loadings, and intercepts but also in residuals across groups, was not supported in this study.

### Bivariate correlations

The associations among the subscales of the PELS were robust, particularly between PELS Coaching and PELS Participation (*r* = 0.76 for GER; *r* = 0.84 for US). Strong positive associations were observed between the PELS and the ELQ across samples (*r* = 0.91 for GER; *r* = 0.94 for US), speaking in favor of *H1*. Additionally, the PELS showed high positive associations with psychological empowerment (*r* = 0.52 for GER; *r* = 0.66 for US), speaking in favor of *H2*. The PELS showed positive associations with job satisfaction (*r* = 0.58 for GER; *r* = 0.67 for US) and negative associations with emotional exhaustion (*r* = −0.42 for GER; *r* = −0.45 for US), speaking in favor of *H3* and *H4, respectively.* These findings are consistent with the expectation that empowering leadership contributes to more positive work situations and fewer negative emotions. Furthermore, a positive association between PELS and job performance (*r* = 0.36 for GER; *r* = 0.55 for US) speaking in favor of *H5*. An overview of all bivariate correlations in Study 1 is given in Table 4.

**Table 4:** Bivariate correlations of the German and US sample – Study 1.

Variable	1	2	3	4	5	6	7	8	9	10	11	12	13
1. Age	—	−0.09	−0.06	−0.15	−0.08	−0.02	−0.012	−0.05	−0.063	0.02	−0.16	0.05	0.09
2. PELS	0.32	—	0.87	0.92	0.92	0.85	0.94	0.87	0.94	0.68	−0.45	0.67	0.55
3. PELS Sense	−0.18	0.85	—	0.73	0.77	0.65	0.84	0.64	0.77	0.64	−0.40	0.59	0.53
4. PELS Coach	0.12	0.90	0.68	—	0.84	0.76	0.83	0.80	0.87	0.56	−0.38	0.56	0.46
5. PELS Part	0.00	0.87	0.70	0.76	—	0.73	0.84	0.79	0.88	0.60	−0.44	0.62	0.49
6. PELS Power	0.53	0.87	0.67	0.75	0.71	—	0.78	0.71	0.79	0.67	−0.36	0.58	0.49
7. PELS Competence	0.28	0.91	0.80	0.78	0.75	0.77	—	0.76	0.87	0.67	−0.43	0.65	0.53
8. PELS Lead	0.48	0.84	0.61	0.74	0.69	0.69	0.69	—	0.88	0.54	−0.41	0.61	0.47
9. ELQ	−0.10	0.91	0.73	0.84	0.83	0.77	0.82	0.79	—	0.61	−0.46	0.66	0.52
10. Empower	0.49	0.52	0.44	0.45	0.44	0.57	0.44	0.40	0.42	—	−0.44	0.71	0.72
11. Exhaustion	0.29	−0.42	−0.35	−0.39	−0.39	−0.34	−0.38	−0.36	−0.41	−0.32	—	−0.62	−0.31
12. Job Satisfaction	0.12	0.58	0.53	0.48	0.49	0.53	0.55	0.46	0.51	0.66	−0.45	—	0.59
13. Performance	0.00	0.36	0.27	0.32	0.29	0.37	0.32	0.32	0.37	0.47	−0.23	0.35	—

Below the diagonal, German samples; above the diagonal, US sample; PELS, psychological empowerment scale; PELS sense, sense making; PELS coach, coaching; PELS part, participation; PELS power, transfer of power; PELS competence, competence development; PELS lead, leading by example; ELQ, empowerment leadership questionnaire; Empower, empowerment; Exhaustion, emotional exhaustion; all correlations |0.12| are significant at *p* < 0.05, correlations |0.15| are significant at *p* < 0.01, and correlations |0.18| are significant at *p* < 0.001.

## Study 2: measuring the effects of PELS over time

To enhance the rigor of our methodology and to improve the quality of our results compared to Study 1, we collected in Study 2 data at two different time points and used ELS instead of ELQ as another additional leadership questionnaire. This approach not only assesses the predictive validity of PELS for future outcomes but also allows us to replicate a selection of our hypotheses over time. To minimize participant dropout, the second measurement point was kept as short as possible. We therefore decided to assess psychological empowerment as a criterion as well as one affective variable and one performance-related variable. These hypotheses build on those derived in Study 1 and are adjusted to reflect the longitudinal nature of the data collection in Study 2, and are as follows:

*H1:* PELS is positively correlated with ELS.

*H2b:* PELS at time 1 correlates positively with later psychological empowerment at time 2

*H3b*: PELS at time 1 correlates positively with later job satisfaction at time 2

*H6b:* PELS at time 1 correlates positively with later innovative behavior at time 2

## Method Study 2

### Procedure

At measurement point 1 (t1), we assessed demographic variables, empowering leadership (using both PELS and ELS), and psychological empowerment. Six weeks later, at measurement point 2 (t2), we again assessed empowering leadership and psychological empowerment. Additionally, we assessed job satisfaction and innovative behavior.

We employed the same data cleaning procedures as in Study 1. Our initial sample included 348 participants at t1 and 264 participants at t2. After matching participants who completed our survey at both measurement points, the final sample size was 175.

### Participants

We recruited participants for our study through social media and personal networks. Upon entering the study, participants completed our study variables and provided their email addresses, which were solely used to invite them for the second measurement wave six weeks later. Participation was entirely voluntary, and participants could withdraw from the study at any time without providing a reason and without facing any negative consequences. To ensure privacy, personal data was handled with strict confidentiality, and email addresses were used exclusively for follow-up purposes. Participants who completed both questionnaires received compensation in the form of personalized feedback on their individual psychological empowerment.

This study assesses demographic and employment characteristics within a diverse workforce, focusing on both employees and their leaders, with the latter’s data reported by the participants. The sample consisted of 175 individuals with a mean age of 44.05 years (*SD* = 9.89), comprising 52 men (29.71%), 121 women (69.1%), and 2 non-binary individuals (1.1%). The leaders, as reported by these participants, included 175 individuals with a mean age of 48.90 years (*SD* = 8.75), primarily men (104, 59.4%) and 71 women (40.6%).

On average, participants have been working 3.99 years (*SD* = 4.41) with their current leader, within teams with a median size of 10 and in organizations having a median size of 1,000 employees. The participants worked an average of 36.68 weekly hours (*SD* = 5.31), across various sectors, including consulting (12.0%), industry and manufacturing (13.1%), and education (16.6%). The sample includes individuals in professional and managerial positions, many of whom report direct and ongoing interactions with their supervisors. This role diversity enables a meaningful assessment of leadership behaviors across hierarchical levels. In addition, the inclusion of participants from sectors with both stable and dynamic team environments support the broader generalizability of the findings. Of the participants, 39.4% worked in more than one team. The sample was highly educated with 87.3% of the participants holding a college degree or higher. Most participants were employees (53.14%), while another 40% held leadership responsibility.

### Materials

We assessed psychological empowerment, job satisfaction, and the demographic variables using the same methodology as in Study 1. Cronbach’s alpha for psychological empowerment was 0.89 at t1 and 0.89 at t2, while McDonald’s omega ω*_t_* was 0.88 at t1 and 0.89 at t2. Cronbach’s alpha for job satisfaction at t2 was 0.80, while McDonald’s omega ω*_t_* was 0.79.

#### Empowering leadership

As in Study 1, we used the newly developed and validated PELS to assess empowering leadership at both t1 and t2. Cronbach’s alpha was 0.96 at t1 and 0.97 at t2, while McDonald’s omega ω*_t_* was 0.96 at t1 and 0.97 at t2. To further establish the validity with older instruments, we also included a German translation of the Empowerment Leadership Scale (ELS; Amundsen and Martinsen, 2014) at t1. Participants rated the frequency of their leader’s empowering leadership behaviors on a scale ranging from 1 (*never*) to 7 (*always*), evaluating 18 statements such as “My leader conveys that I shall take responsibility.” Cronbach’s alpha at t1 was 0.94, while McDonald’s omega ω*_t_* was 0.94.

#### Innovative behavior

We used nine items adapted from Janssen (2000) to assess innovative behavior. Participants rated the frequency of their innovative actions on a scale from 1 (*never*) to 5 (*always*). For example, participants responded to statements like “How often do you create new ideas for difficult issues?” Cronbach’s alpha at t2 was 0.90, while McDonald’s omega ω*_t_* was 0.90.

## Results Study 2

### Confirmatory factor analyses

For Study 2, the PELS scale’s model fit was assessed at two time points. At time 1, the model fit indices were CFI = 0.93 and RMSE*A* = 0.072, improving at time 2 to CFI = 0.96 and RMSE*A* = 0.061. Conversely, the Empowering Leadership Scale (ELS) at time 1 presented a lower fit with a CFI of 0.83 and an RMSEA of 0.129, suggesting issues with the scale’s structural validity. An overview of all measurement models in Study 2 is given in Table 5.

**Table 5:** Measurement models in Study 2 using MLR^a^ estimator.

Variable	*N* factors	*Χ*^2^	df	*p*	CFI	TLI	SRMR	RMSEA	RMSEA 90% CI
PELS									
T1	6 + g	626.91	246	<0.001	0.93	0.92	0.054	0.072	0.065–0.079
T2	6 + g	460.02	246	<0.001	0.96	0.95	0.051	0.061	0.052–0.070
ELS									
T1	2	782.93	134	<0.001	0.83	0.80	0.070	0.129	0.120–0.138
Empowerment									
T1	4 + g	81.56	50	0.003	0.99	0.98	0.041	0.049	0.028–0.068
T2	4 + g	68.45	50	0.043	0.99	0.99	0.036	0.040	0.008–0.062
Innovation									
T2	3	25.68	24	0.37	1.00	1.00	0.024	0.017	0.000–0.057

*N* = 175; ^a^Maximum likelihood with robust standard errors; PELS, Psychological Empowerment Leadership Scale; ELS, empowering leadership scale; T1, first data collection; T2 = 6 weeks follow-up data collection.

### Stability and criterion validity

The PELS showed strong stability over time (*r* = 0.83 between t1 and t2). The PELS demonstrated also strong positive correlations with the ELS (*r* = 0.91 at t1, *r* = 0.76 at t2) and psychological empowerment (*r* = 0.43 at t1, *r* = 0.39 at t2), supporting the associations expected in Hypotheses 1 and 2 across both time points. This affirms a consistent positive association between empowering leadership behaviors and psychological empowerment constructs.

A positive correlation was observed between PELS and job satisfaction (*r* = 0.36 at t1, *r* = 0.52 at t2), consistent with Hypotheses 3. The correlation suggests that higher scores on empowering leadership are associated with increased job satisfaction. The expected positive correlation between PELS and innovative behavior was not statistically significant (*r* = 0.07 at t2). This indicates that the association between empowering leadership and innovative behavior, as measured in this study, was not established, thus speaking against *H6*. But the same was true for ELS (*r* = 0.09 at t2). An overview of all bivariate correlations in Study 2 is given in Table 6.

**Table 6:** Bivariate correlations Study 2.

Variable	1	2	3	4	5	6	7	8	9	10	11	12	13
1. Age	1.00												
2. PELS	−0.11	1.00											
3. PELS sense	−0.06	0.85	1.00										
4. PELS coach	−0.09	0.90	0.69	1.00									
5. PELS part	−0.04	0.81	0.58	0.73	1.00								
6. PELS power	−0.17	0.82	0.61	0.73	0.71	1.00							
7. PELS competence	−0.14	0.88	0.80	0.74	0.60	0.65	1.00						
8. PELS lead	−0.04	0.77	0.55	0.66	0.53	0.51	0.59	1.00					
9. ELS	−0.12	0.91	0.75	0.80	0.73	0.76	0.82	0.69	1.00				
10. Empower	0.13	0.43	0.37	0.34	0.40	0.53	0.35	0.19	0.41	1.00			
11. PELS T2	−0.10	0.83	0.71	0.74	0.68	0.69	0.70	0.67	0.76	0.39	1.00		
12. Empower T2	0.12	0.35	0.27	0.30	0.38	0.46	0.26	0.12	0.29	0.83	0.41	1.00	
13. Job satisfaction T2	0.25	0.36	0.36	0.33	0.32	0.33	0.33	0.14	0.29	0.52	0.36	0.59	1.00
14. Innovation behavior T2	0.10	0.07	0.07	0.05	0.12	0.15	0.04	−0.04	0.09	0.42	0.17	0.43	0.14

*N* = 175; PELS, psychological empowerment scale; PELS Sense, sense making; PELS coach, coaching; PELS part, participation; PELS power, transfer of power; PELS competence, competence development; PELS lead, leading by example; ELS, empowerment leadership scale; Empower, empowerment; T2 = 6 week follow up; all correlations |0.17| are significant at *p* < 0.05, and correlations |0.25| are significant at *p* < 0.001.

## Discussion

This study sought to develop and validate the PELS, an instrument designed to assess the dimensions of empowering leadership within the frameworks of psychological empowerment. We consider a new instrument to be important due to the limitations of existing tools, which are not sufficiently embedded in the empowerment literature. We will begin by discussing the results of the validation efforts for the PELS.

The results from the confirmatory factor analyses provided support for the factorial validity of the PELS. Both the German and U. S. samples demonstrated good model fit, indicating that the scale effectively captures the empowering leadership construct across different cultural contexts. Furthermore, our analyses revealed metric measurement invariance with good model fit between Germany and the US. These results suggest that researchers can compare correlations using the PELS across German and US samples. Although full support for scalar invariance was not achieved, the deviations were only marginal (ΔCFI = −0.010, ΔRMSE*A* = 0.006), and the model fit for the scalar measurement model remained acceptable. Therefore, we suggest that comparing means between these groups is feasible. However, scholars should interpret these results cautiously and consider conducting their own measurement invariance analyses as needed. In summary, our findings suggest that the PELS is a robust tool that can be reliably used in both countries.

Regarding the associations between empowering leadership and work-related outcomes, our findings support the criterion validity of the PELS. The scale correlated positively with psychological empowerment in both countries and predicted it over time. These results align with prior research suggesting empowering leadership can enhance employees’ sense of control and motivation (Spreitzer, 1995). Notably, the power dimension of the PELS was particularly robust, confirming its theoretical centrality. This result is especially noteworthy, as it is precisely this dimension that is explicitly missing in older instruments for measuring empowering leadership.

Moreover, the PELS demonstrates robust associations with job satisfaction (Study 1: *r* = 0.58 and 0.67; Study 2: *r* = 0.36) and emotional exhaustion (Study 1: *r* = −0.42 and −0.45), indicating that empowering leadership contributes positively to workplace satisfaction and reduces emotional strain among employees. This is in line, for example, with the Demand-Control Model (DCM; Karasek, 1979), which predicts that people with more control are less strained. Notably, the correlations observed in our studies exceed those reported in the meta-analysis (*r* = 0.38 for job satisfaction; *r* = −0.19 for emotional exhaustion; Kim et al., 2018), often showing correlations that are twice as strong.

Similarly, the association between empowering leadership and job performance was in line with prior findings (Kim et al., 2018). However, the correlation between the PELS and innovative behaviors was lower than expected based on meta-analytic results. This pattern is consistent with findings for the ELS, suggesting that such outcomes may not be as directly linked to empowerment-oriented leadership as previously assumed. One possible explanation lies in how innovation was conceptualized in prior research. For example, the meta-analysis by Kim et al. (2018) combined innovative behavior and creativity, which may conflate distinct constructs—implementation versus idea generation. The definition by Basu and Green (1997) emphasizes both novelty and execution, and it is possible that studies with a stronger focus on creativity inflated the overall effect size. Additionally, sample characteristics in Study 2 may have played a role: nearly 40% of participants worked in multiple team settings, which may diffuse consistent innovation behavior tied to one leader. It is also plausible that the link between empowering leadership and innovation is mediated by other factors, such as psychological safety or team climate. Future research should explore these dynamics more closely to clarify under which conditions empowering leadership supports innovative behavior.

Finally, it is important to compare the PELS with the existing instruments. Theoretically, the PELS holds some advantages over the ELQ and ELS because the dimensions have been deductively derived from the empowerment literature. In particular, the literature relating to psychological empowerment (Spreitzer, 1995) was integrated into the development of the PELS, ensuring that the tool is deeply grounded in the relevant literature. This connection to psychological empowerment theory distinguishes the PELS from legacy instruments that were based more on exploratory factor structures than theoretical grounding. This strong theoretical foundation enhances the PELS’s utility in measuring and understanding empowerment processes, providing a more comprehensive and nuanced approach compared to its predecessors.

From an empirical perspective, the PELS demonstrates the same high reliability, with Cronbach’s alpha and McDonald’s omega consistently above 0.90, comparable to the ELQ and ELS. However, one notable advantage is that we present information on the stability of the PELS with a correlation of *r* = 0.83 between t1 and t2. This indicates that it reliably assesses empowerment-oriented leadership behavior over time, assessing stable aspects of managerial behavior that are less influenced by situational factors or temporal variation. As expected, correlations between the PELS and ELQ/ELS indicate strong concurrent validity, suggesting that these instruments measure similar constructs. This positions the PELS well into the current landscape of available instruments to assess empowering leadership. However, the PELS offers several advantages: it shows a better model fit in confirmatory factor analyses and encompasses a broader theoretical content compared to the ELQ and ELS. Moreover, the PELS achieves these results with a set of only 24 items, which is 14 fewer than the ELQ. Thus, it offers an efficient option for studying leadership behavior.

### Practical implications

With the PELS it seems possible to assess empowering leadership in an organizational context in a reliable and valid way. This enables managers and organizations to generate information on how empowering the behavior of their managers is perceived. This information can be used, for example, as part of a 360-degree feedback, where supervisors receive comprehensive feedback on their leadership behavior. Organizations can systematically identify areas for leadership development and provide targeted training. This ensures that leadership development efforts are directly aligned with the actual needs and perceptions of employees. Furthermore, if empowering leadership is used as a leadership model within an organization, the PELS serves as an effective means to evaluate how well this model is implemented by the leaders and perceived by the employees.

In addition to the stronger theoretical foundation, the PELS also offers several practical advantages. Firstly, with 24 items, the PELS allows for a relatively quick assessment of empowering leadership, making it suitable for regular use in organizational settings without requiring extensive time commitments (e.g., pulse check ins). Secondly, the PELS consists of six facets each demonstrating robust reliability. Using distinct facets enables a more nuanced and differentiated feedback, allowing organizations to identify specific areas of empowering leadership that may require attention.

### Limitations

This study faces several limitations that may affect the generalizability and interpretation of the findings. First, the recruitment of participants in Germany through personal and professional social networks led to a non-probability sample. Although this method likely improved response rates and participant diversity, it limits the ability to generalize these findings broadly. Second, both samples were drawn from Western cultures (Germany and the USA), raising concerns about the generalizability of the scale to non-Western cultures. Cultural differences may influence perceptions of leadership behavior, so further research is needed to validate the instrument in diverse cultural contexts.

Another limitation is the reliance on self-reported data, which can introduce biases such as social desirability or inaccuracies in self-perception. Although self-report methods are often critiqued for potential bias, some researchers argue that self-report methods might not be inherently flawed (Chan, 2009). Furthermore, since all variables were assessed through self-reports, this raises concerns about potential common method bias (Podsakoff et al., 2003). Yet, literature suggests that the impact of common method bias on correlation might be overstated (e.g., Bozionelos and Simmering, 2022; Spector and Brannick, 2010).

Lastly, the construct validation for the tools adapted for German- and English-speaking populations was conducted strictly within Germany and the USA. Subtle linguistic and cultural differences could affect the validity of the instrument. To ensure the instrument’s validity in other German-speaking regions, such as Austria and certain parts of Switzerland, or in other English-speaking areas, including the United Kingdom, Canada, and Australia, further validation is necessary.

## Conclusion

This research introduces the Psychological Empowerment Leadership Scale (PELS) as a theory-driven, psychometrically sound instrument that directly aligns empowering leadership behaviors with the dimensions of psychological empowerment. Across two studies and two cultural contexts, the PELS demonstrated strong factorial validity, reliability, and predictive power. Its alignment with a robust theoretical framework distinguishes it from existing measures and offers both scientific and practical benefits. By enabling a more precise assessment of empowerment-oriented leadership, the PELS contributes to advancing leadership research and provides organizations with a useful tool for leadership development and feedback. Future research should explore its use across diverse cultures and in longitudinal designs to further investigate its role in promoting sustainable and empowering work environments.

## Data Availability

The raw data supporting the conclusions of this article will be made available by the authors, without undue reservation.
